# Progress in Surgery of the Liver, Gall Bladder, and Spleen

**Published:** 1904-02-20

**Authors:** 


### PROGRESS IN SURGERY OF THE LIVER, GALL BLADDER, AND SPLEEN.

**Operation for the Ascites of Cirrhosis.**—Barker[1] considers that in the ordinary methods of performing Talma's operation, the way in which the anastomosis between the systemic veins and those of the portal system is commonly arrived at is defective. He therefore operated in the following manner:—The abdomen was opened on the mid-line below the navel. When the ascitic fluid had run off, the omentum, which was very thick and sclerosed, was drawn out and divided down its middle into two long lobes. The thickened and vascular parietal peritoneum was now stripped off from the abdominal wall on both sides of the median incision over an area about the size of the hand. In the pockets so formed a transverse cut was made above in each case for about 1½ inches. Through the holes thus formed each lobe of the omentum was passed into the pockets on either side, spread out all over the pocket, and secured to its lowest point by one or two sutures. The abdominal wound was then closed in the usual manner. What was hoped was this, namely, that the two portions of omentum, being now covered by raw surfaces of vascular connective tissue on both sides, would rapidly become incorporated with the abdominal wall. Such adhesions between serous surfaces and connective tissue are known to be permanent. White[2] endeavoured to achieve the same object by detaching and turning back the peritoneum around the wound. The stripped area would be about 24 square inches in extent, and against this the anterior surface of the great omentum was fixed by half-a dozen sutures of fine silk. He records two cases and Sheen[3] records another.

**Partial Hepatectomy.**—Lockwood[4] has removed Riedel's lobe of the liver in a case in which there appeared no other cause for the pain which the patient suffered. The swelling before operation was supposed to be a movable kidney. He considers that the liver is singularly tolerant of surgical interference and that hæmorrhage from its substance is easily controlled and not to be dreaded. Cripps[5] removed a large nævoid tumour of the liver from a man aged 60 years. The parietal peritoneum was slightly adherent over the surface of the swelling. On separating this a small opening was made into it, and a stream of fairly bright blood issued with some force. On examining the swelling, which was about the size of a child's head, it was found to be in the substance of the liver, partly protruding from the under surface, and partly covered by a thin layer of liver substance. Partly by dissecting and partly by a Volkmann's spoon the entire tumour was nucleated. The bleeding, which was sharp at first, was easily controlled by sponge pressure. The abdominal walls were closed without drainage and the patient made a good recovery.

**Operations on the Gall-bladder.**—Eve[6] reports three cases of cholecystectomy, and considers that the indications for this operation are most evident when gall-stones are so firmly impacted in the cystic duct that they cannot be dislodged. The stones may be removed by incision and suture of the duct (Robson). The duct may, however, be ulcerated where the stone lay or there may be a cicatricial condition above it, both being conditions likely to give rise to future trouble. Further, the operation of suture of the duct and subsequently of the wound in the gall-bladder to the parietes is likely to be more prolonged and more difficult than cholecystectomy. Gibbon[7] says gangrenous cholecystitis is a condition of extreme rarity. It appears to be induced by obstruction of the cystic duct and infection by virulent organisms. It is doubtful if gangrene of the gall-bladder could develop with a patulous cystic duct. The impacted stone probably acts in the same way as an enterolith does in gangrenous appendicitis. Precisely the same treatment should apply to a gangrenous gall-bladder as applies to a gangrenous appendix. Simple drainage is not recommended ; the gall-bladder should be removed, and the duct drained. Bidwell[8] says the symptoms of acute phlegmonous cholecystitis are usually as follows :—A high temperature of 103° Fahr. or over, a rapid feeble pulse, quick and shallow breathing, intense depression, an anxious expression, and persistent vomiting. Occasionally severe diarrhœa is also present. Locally the abdomen will be found to be distended and tympanitic, there will be acute tenderness in the region of the gall-bladder, extending perhaps to the right iliac region, and the abdominal walls will not move on respiration ; in fact, the condition will be one of acute peritonitis. In some fortunate cases the pus may become localised precisely in the same way as it does in appendicitis ; otherwise the prognosis is most grave, even when the operation is undertaken before any purulent collection has taken place within the peritoneal cavity. Suppurative catarrh of the gall-bladder is much less serious; but if no operation is performed the gall-bladder may perforate after the formation of adhesions, and an abscess may come to the surface between the layers of the abdominal wall or more commonly at the umbilicus. In other cases the gall-bladder may form adhesions between the colon and small intestine and the gall-stones and pus may be discharged into the intestinal canal. In other cases again, no inflammation is excited outside the gall-bladder, but the tumour remains and the patient becomes emaciated.

**Rupture of the Spleen.**—The symptoms to which laceration of the spleen gives rise are those of internal hæmorrhage, but the rapidity of the onset varies greatly in different cases.[9] The diagnosis is often not difficult ; the signs of internal hæmorrhage are marked, both flanks are dull on percussion, but only the right flank becomes completely resonant on change of position. This immovable dulness in the left flank is due to the great masses of clot which surround the torn spleen. As to the treatment of ruptured spleen when the diagnosis has been made, there cannot be two opinions. Operation is indicated, and it should be commenced at once in the judgment of most surgeons. The best incision for removal of a ruptured spleen is in the left semilunar line, commencing at the edge of the ribs ; its length must depend on the size of the spleen, but in cases where the viscus is normal in size four inches should be sufficient. Where the diagnosis is uncertain, a preliminary central incision is advisable, to be followed by the lateral incision when the diagnosis is established. The spleen is brought to the wound, its pedicle is securely ligatured, and the viscus is removed. No hesitation need be felt in removing a ruptured spleen. Most patients with rupture of the spleen recover rapidly after excision, though convalescence may be retarded by a peculiar form of weakness, probably due to delay in the establishment of the vicarious functions of the lymph glands and the bone marrow.

1 Edinburgh Med. Jour., July, 1903, p. 57. 2 Brit. Med. Jour., Oct. 19, 1903, p. 898. 3 Ibid., p. 899. 4 Lancet, July 25, 1903, p. 223. 5 Brit. Med. Jour., July 4, 1903, p. 28. 6 Lancet, June 27, 1903, p. 1808. 7 Edinburgh Med. Jour., July, 1903, p. 73. 8 Lancet, Aug. 29, 1903, p. 586. 9 Lancet, Sept. 26, 1903, p. 895.

